# Comparative analysis of Foxp2 expression in the thalamus of mice, rats, and macaques: implications for the evolution of language circuits

**DOI:** 10.1007/s00429-026-03165-x

**Published:** 2026-07-24

**Authors:** Blanca Sánchez-Moreno, Alicia Uceda-Heras, María de la Fuente-Fernández, Miguel Ángel García-Cabezas, Carmen Cavada, Javier Gilabert-Juan

**Affiliations:** PhD Program in Neuroscience, Universidad Autónoma de Madrid-Cajal Institute, Department of Anatomy, Histology, and Neuroscience, School of Medicine, Universidad Autonoma de Madrid, Madrid, Spain

**Keywords:** Foxp2, Thalamus, Language circuits, Comparative neurology, Evolutionary changes

## Abstract

Foxp2 is a transcription factor essential for the development and function of neural circuits involved in language. Although its expression has been extensively characterized in the cortex and basal ganglia, its organization within the adult thalamus remains poorly understood. In this study, we present a comparative analysis of Foxp2 protein expression across thalamic nuclei in mice, rats, and macaques, with a focus on nuclei associated with higher-order cognitive functions and language-related circuits in humans. We found that Foxp2 is expressed in most thalamic nuclei across species, with a consistent absence in the reticular nucleus and zona incerta. Expression was highest in midline and intralaminar nuclei, whereas the anterior group showed low and variable expression among species. Macaques exhibited broader and, in some nuclei, more intense FoxP2 expression, particularly in associative regions such as the pulvinar and parts of the ventral group, consistent with differences in thalamocortical organization across species. This distribution suggests a conserved role for Foxp2 in shaping thalamic circuits supporting sensorimotor integration, attention, memory, and linguistic processing. Phylogenetic comparisons suggest that enhanced Foxp2 expression in associative thalamic territories in primates may be related to the increased complexity of thalamocortical circuits. These findings provide molecular and anatomical insights into the organization of thalamocortical networks and may offer a framework for understanding their involvement in higher-order cognitive functions and neuropsychiatric disorders.

**Supplementary Information:**

The online version contains supplementary material available at https://doi.org/10.1007/s00429-026-03165-x.

## Introduction

FOXP2 is a transcription factor belonging to the forkhead box (FOX) family, characterized by a conserved forkhead DNA-binding domain and additional regions mediating dimerization and transcriptional regulation (Lai et al. 2001; Shu et al. 2001). As a nuclear protein, FOXP2 regulates the expression of a wide range of target genes involved in neuronal differentiation, neurite outgrowth, and synaptic plasticity (den Hoed et al. 2021). Mutations in *FOXP2* were first described in the KE family (Lai et al. 2001), in which affected members exhibited developmental verbal dyspraxia, marked by deficits in speech motor control, language processing, and grammatical skills (Vargha-Khadem et al. 1998). These findings positioned FOXP2 as a key molecular entry point into the genetic and neurobiological foundations of language. FOXP2 is highly expressed in distinct neuronal populations within cortico-striato-thalamo-cortical circuits, suggesting a conserved role in motor sequencing and sensorimotor integration (Fisher and Scharff 2009; Vargha-Khadem et al. 2005).

The thalamus is a critical hub supporting both linguistic processing and broader cognitive functions (Klostermann et al. 2013). Ischemic or hemorrhagic lesions can result in thalamic aphasia (Fritsch et al. 2022), underscoring its involvement in speech and language. Although the specific contribution of individual thalamic nuclei remains incompletely understood, the ventral anterior (VA), ventrolateral (VL), intralaminar, and pulvinar nuclei have been most consistently implicated in language functions (Barbas et al. 2013; Crosson 2013, 2021a; Llano 2013). Other nuclei, such as the reticular, mediodorsal (MD), and midline nuclei, are thought to support language indirectly through their roles in memory, attention, and executive functions (Crosson 2013; Perea-Bartolomé and Ladera-Fernández 2004). Together, these findings highlight the thalamus as a dynamic integrator within the distributed neural network that underlies speech and linguistic cognition.

Within this context, FOXP2 expression in the thalamus becomes particularly relevant. FOXP2 is expressed from early embryonic stages (Alhesain et al. 2025; Lai et al. 2003), displaying a conserved spatial and temporal pattern that persists into adulthood with varying intensity across different nuclei (Lai et al. 2003; Takahashi et al. 2008). Functional studies indicate that FOXP2 contributes to thalamic patterning and the specification of regional thalamic identities, guiding the formation and refinement of thalamocortical projections (Alhesain et al. 2025; Ebisu et al. 2017). Through these mechanisms, FOXP2 may influence the development of the very circuits that support word finding, speech sequencing, and the integration of attention and memory during language tasks. Despite these advances, the specific distribution of FOXP2 expression within the mature thalamus remains poorly characterized. Most research has focused on cortical and striatal regions, leaving the molecular organization of thalamic language-related circuits largely unexplored.

In this study, we present a comparative analysis of Foxp2 protein expression in the thalamus of mice, rats, and macaques, focusing on nuclei associated with higher-order cognitive functions, including those linked to language in humans. By examining both the nuclear distribution of Foxp2 and the thalamocortical projection patterns of these nuclei, this work aims to relate the expression profiles of this transcription factor to the functional architecture of thalamic circuits, particularly those involved in higher-order cognitive processing, within a comparative and evolutionary framework.

## Materials and methods

### Animals

Tissue from three macaques (two males of *Macaca Mulatta* and one female of *Macaca nemestrina)*, three male rats (*Rattus norvergicus*; Wistar) and three male mice (*Mus musculus*; C57BL/6) was used to study the distribution of Foxp2 in the thalamus. All animals used were adults housed under controlled temperature and a 12-h light/dark cycle with food and water available ad libitum. The ethics protocol under which the experiments were approved was PROEX 209.7/22 and CEI-39–852. All animal experimentation was conducted in accordance with Directive 2010/63/EU of the European Parliament and of the Council of 22 September 2010 on the protection of animals used for scientific purposes and was approved by the Committee on Bioethics of the Universidad Autónoma de Madrid. Every effort was made to minimize the number of animals used and their suffering.

Animals were deeply anesthetized with pentobarbital (Dolethal, Vetoquinol) and perfused transcardially with a 4% paraformaldehyde (Panreac) solution in phosphate buffer (PB; 0.1 M, pH 7.4). Brains were then extracted, cryoprotected in a 30% sucrose solution and sectioned coronally at 50 μm using a sliding microtome for rodents and 40 μm coronal sections for macaques. 10 series were collected from rat and macaque brains, and 4 series were collected from mice brains. One series from each was processed for acetylcholinesterase histochemistry and another for cresyl-violet staining to identify thalamic nuclei. A full series was used for Foxp2 immunohistochemistry in mouse and rat brains. In macaques, sections spanning the entire thalamus were selected to maximize representation of all thalamic nuclei; therefore, sampling did not follow a fixed inter-section spacing.

### Immunohistochemistry

Immunohistochemistry was performed on free-floating macaque brain sections as follows: Sections were washed three times for 10 min each in TBS 0.1 M, followed by two 10-min washes with endogenous peroxidase inactivation solution (TBS with 3% H2O2 and 10% methanol). After three additional 10-min washes, antigen retrieval was performed in a 10 mM sodium citrate buffer (pH 6.0) for 45 min at 80 °C. Sections were then washed three times for 5 min and blocked for 4 h at 4ºC with TBS containing 20% normal goat serum (NGS, Biowest), 5% bovine serum albumin (BSA, Sigma-Aldrich) and 0.4% Triton X-100, then incubated with the primary antibody mouse anti-Foxp2 (1:500, ThermoFisher, CL5312) in the same buffer containing 0.2% Triton X-100 three overnights at 4ºC. Following three additional 10-min washes, the secondary antibody goat anti-mouse (1:200, Merck, AP181B) was applied for 3 h at room temperature, followed by three 10-min washes and incubation in avidin–biotin complex for 1 h. Labeling was visualized with 3,3′-diaminobenzidine (DAB, Sigma-Aldrich) and H₂O₂, and after three 10 min washes in 0.1 M phosphate buffer (PB), sections were mounted in 0.033 M PB. Sections were then dehydrated through graded ethanol solutions (50%, 70%, 80%, 90%, 100%), cleared through five successive changes of xylene and coverslipped with DEPEX (SERVA Electrophoresis GmbH).

For rat and mouse sections, the same protocol was used with the following modifications: Washes were performed using 0.1 M PBS with 1% Triton X-100; antigen retrieval was shortened to 15 min; blocking solution contained 10% NGS in PBS with 1% Triton X-100; incubation buffer contained 3% NGS in PBS with 1% Triton X-100; primary antibodies were incubated overnight, and secondary antibodies for 1 h.

Immunohistochemical protocols were optimized separately for rodents and macaques in order to achieve adequate antibody penetration and signal-to-noise ratio. This limits the comparability of absolute staining intensity across species. In combination with species‑specific differences in thalamic cell density and a small sample size (n = 3 per species), this makes formal statistical comparisons between species difficult. Therefore, the present analysis focuses on semiquantitative and descriptive patterns of Foxp2 expression.

### Image preparation and thalamic nuclei delimitation

Images of Foxp2 immunostaining in the thalamus were acquired as mosaics of 20x-objective pictures using Neurolucida software (MBF Bioscience, USA) on a personal computer connected to a Zeiss Axioskop microscope (Zeiss, Germany) through a CX9000 camera (MBF Bioscience, USA) and a motorized microscope stage (MBF Bioscience, USA; Heidenhain Corporation, USA). Image resolution was set to 0.5 pixels/μm for rat and mouse sections and 1 pixel/μm for macaque sections.

Images of AChE and Nissl stainings were obtained in the same manner, using mosaics of 2.5 × objective images. Thalamic nuclei were delineated with reference to The Mouse Brain in Stereotaxic Coordinates (5th Edition) (Paxinos and Franklin 2019), The Rat Brain in Stereotaxic Coordinates (7th Edition) (Paxinos and Watson 2014), The Thalamus of the Macaca Mulatta (Olszewski 1952) and The Rhesus Monkey Brain in Stereotaxic Coordinates (Paxinos et al. 2000).

### Foxp2 + cell density and intensity analysis

Foxp2 + cell density and intensity were measured using ImageJ. To estimate cell density, the thalamic nuclei were outlined to measure their area and count the number of Foxp2 + cells. An average cell density was then calculated for each nucleus. Based on the highest observed cell density, we assigned a density level of 0 to the nuclei with densities less than 5% of this maximum. The remaining range (from 5% to the maximum density) was divided into five equal intervals, and each thalamic nucleus was assigned a density level according to its value.

To assess Foxp2 + cell intensity, between one and three squared Sects. (5.000 μm^2^ for mice, or 20.000 μm^2^ for rats and monkeys) were selected from each thalamic nucleus. These section sizes were chosen to maximize sampling area while ensuring that the regions could be fully contained within the smallest nuclei analyzed in each species. These images were processed using a custom ImageJ macro to calculate the mean gray value of the cells and the background (Supplementary data). A cell-to-background intensity ratio was calculated to account for differences in background across nuclei. An average intensity ratio was determined for each nucleus. Using the highest and lowest intensity values, we divided the range into five equal levels and assigned an intensity level to each thalamic nucleus accordingly. For both density and intensity analyses, measurements were obtained for each thalamic nucleus individually. Relative expression levels were first assigned on a per-animal basis and subsequently integrated to determine species-level expression patterns.

## Results

In this study, cell density reflects the number of Foxp2-positive cells per area unit within each thalamic nucleus, whereas labeling intensity reflects the relative level of Foxp2 expression within individual cells; thus, these measures capture complementary aspects of protein distribution, namely how widespread versus how strong the expression is.

**Fig. 1:**
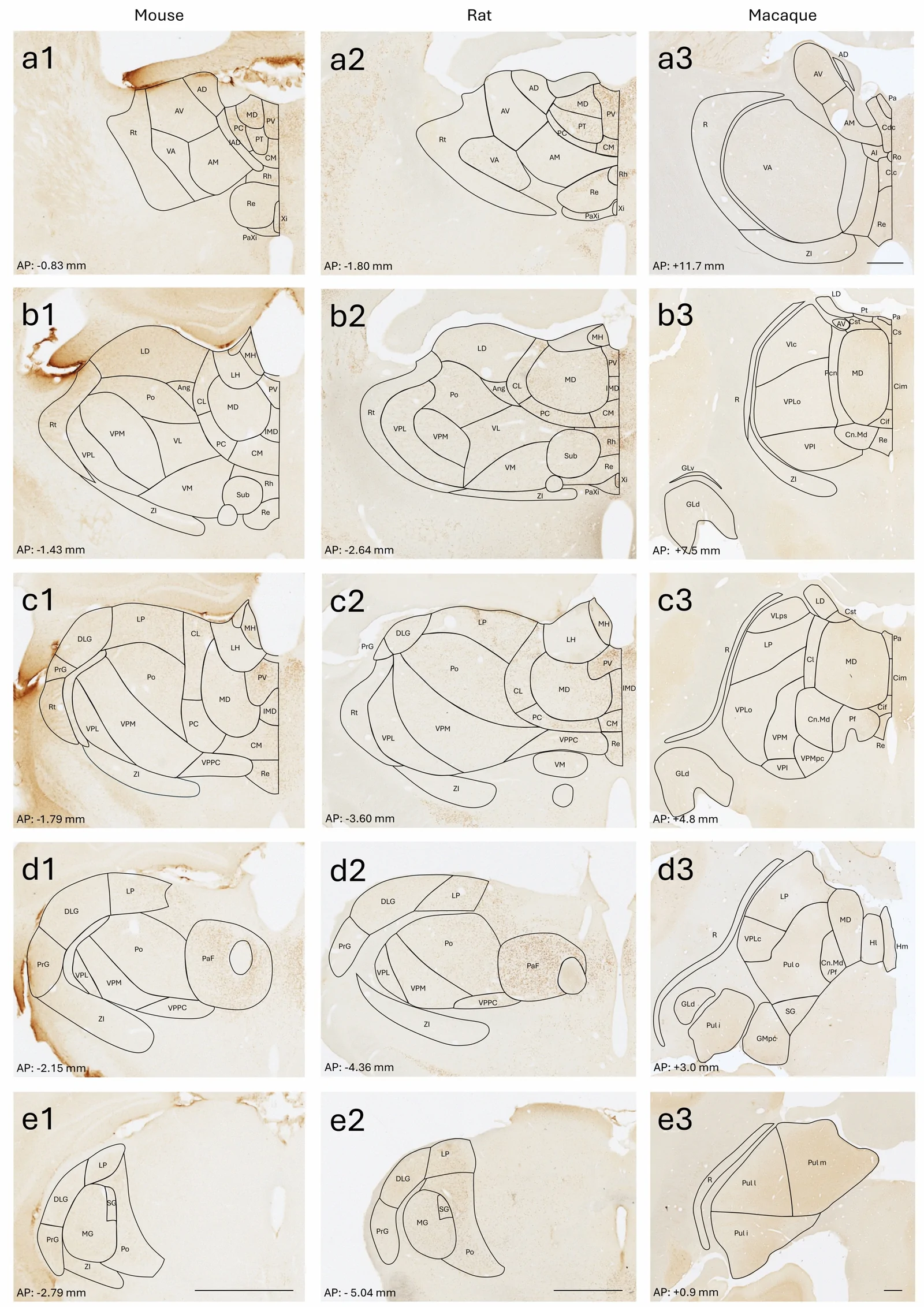
Foxp2 immunohistochemistry in coronal thalamic sections of mice (1), rats (2), and macaques (3). Five representative sections per species, spanning the anterior–posterior axis, are shown. Thalamic nuclei have been delineated. Scale bars: 1.000 μm

**Fig. 2:**
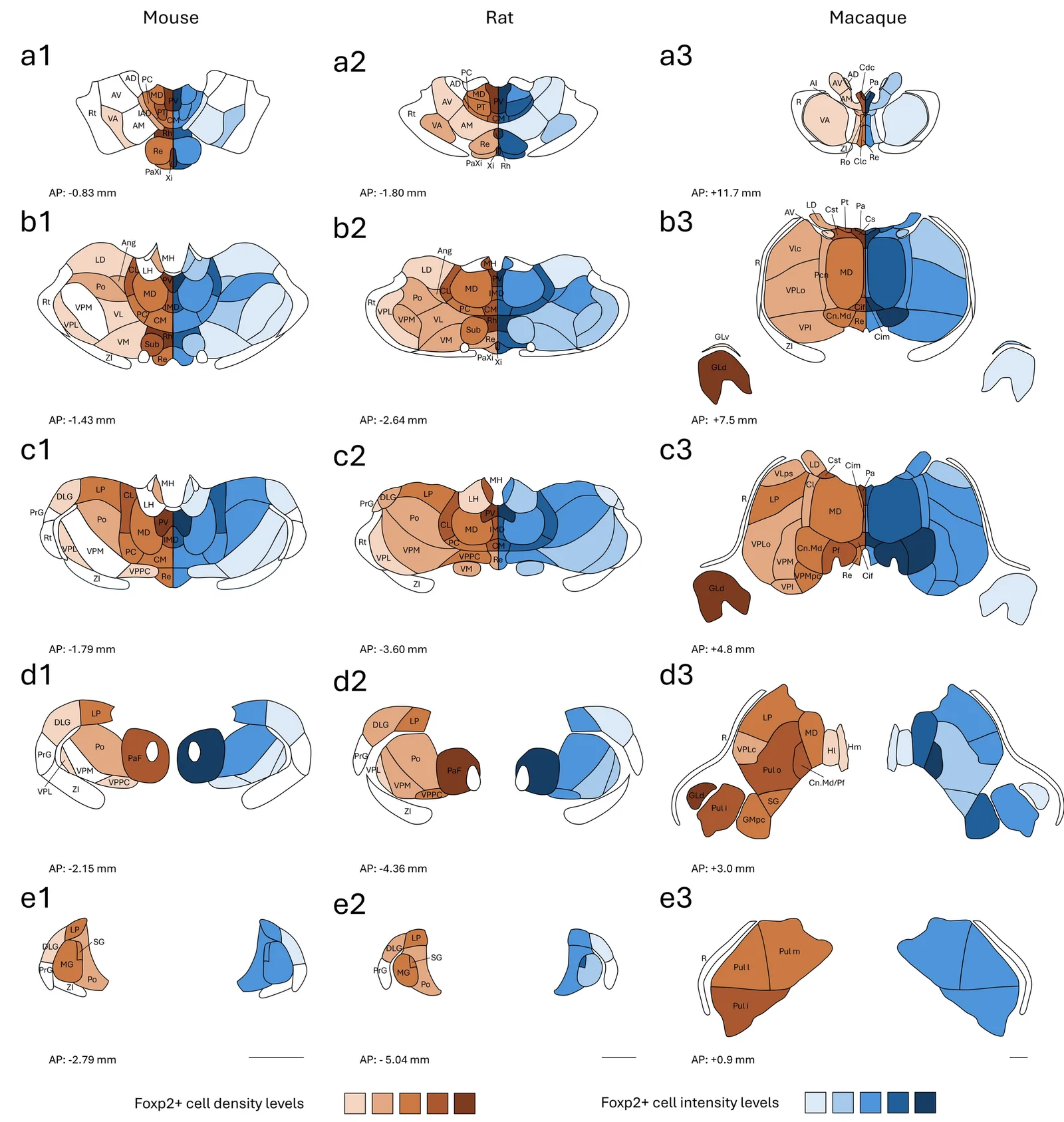
Graphical summary of Foxp2-positive cell density (brown, left) and intensity (blue, right) in coronal thalamic sections of mice (1), rats (2), and macaques (3). Five representative sections per species, spanning the anterior–posterior axis, are shown. Scale bar: 1.000 μm

Overall, Foxp2 expression patterns in the thalamus were relatively conserved across the species studied (Fig. 1). Foxp2 was expressed across all thalamic nuclei of macaques and in almost all thalamic nuclei of rats and mice, except for the reticular (R/Rt) nucleus and the zona incerta (ZI). Expression was highest in the midline and intralaminar nuclei and lower in the anterior group. Below, we describe Foxp2 expression density and intensity across thalamic nuclei. To provide a clearer overview of the results, we present a summary figure depicting Foxp2 cell density and intensity across thalamic nuclei (Fig. 2), as well as summary tables indicating Foxp2 + cell density and intensity across all studied thalamic nuclei, together with the number of sections in which each nucleus was represented (Tables 1, 2, 3). When a nucleus was present in both hemispheres within the same section, each hemisphere was counted separately.

**Table 1:** Summary of FoxP2 cell density and intensity levels and number of analyzed sections for each thalamic nucleus in mice

	Thalamic nucleus	Density	Intensity	N
*Midline group*				
PV	Paraventricular nucleus	5	5	40
IMD	Intermediodorsal nucleus	4	4	26
IAM	Interanteromedial nucleus	2	1	4
CM	Central medial nucleus	3	3	40
Rh	Rhomboid nucleus	5	4	28
Re	Reuniens nucleus	3	3	38
PaXi	Paraxiphoid nucleus	3	4	28
Xi	Xiphoid nucleus	5	5	28
PT	Paratenial nucleus	4	3	6
*Intralaminar group*				
Ang	Angular nucleus	2	2	12
CL	Centrolateral nucleus	4	4	34
PC	Paracentral nucleus	3	3	40
PaF	Parafascicular nucleus	4	5	14
*Anterior group*				
AD	Anterodorsal nucleus	0	0	10
AV	Anteroventral nucleus	0	0	12
AM	Anteromedial nucleus	0	1	12
IAD	Interanteromedial nucleus	2	1	8
LD	Laterodorsal nucleus	1	1	18
*Mediodorsal nucleus*				
MD	Mediodorsal nucleus	3	3	38
*Ventral group*				
VA	Ventral anterior nucleus	1	2	6
VL	Ventrolateral nucleus	1	2	20
VM	Ventromedial nucleus	1	1	34
VPM	Ventral posteromedial nucleus	0	1	41
VPL	Ventral posterolateral nucleus	1	1	42
VPPC	Ventral posterior parvicellular nucleus	1	1	18
Sub	Submedius nucleus	2	2	26
*Posterior group*				
LP	Lateral posterior nucleus	3	3	38
Po	Posterior nucleus	2	3	51
SG	Suprageniculate nucleus	3	3	20
*Geniculate nuclei*				
DLG	Dorsal lateral geniculate nucleus	1	1	37
MG	Medial geniculate nucleus	3	3	22
*Other*				
MH	Medial habenular nucleus	2	2	34
LH	Lateral habenular nucleus	0	2	34
PrG	Pregeniculate nucleus	0	0	42
Rt	Reticular nucleus	0	0	60
ZI	Zona incerta	0	0	33

**Table 2:** Summary of FoxP2 cell density and intensity levels and number of analyzed sections for each thalamic nucleus in rats

	Thalamic nucleus	Density	Intensity	N
*Midline group*				
PV	Paraventricular nucleus	5	5	38
IMD	Intermediodorsal nucleus	4	4	22
IAM	Interanteromedial nucleus	2	3	2
CM	Central medial nucleus	4	4	36
Rh	Rhomboid nucleus	5	5	26
Re	Reuniens nucleus	2	3	36
PaXi	Paraxiphoid nucleus	2	3	20
Xi	Xiphoid nucleus	5	4	14
PT	Paratenial nucleus	3	4	12
*Intralaminar group*				
Ang	Angular nucleus	2	3	8
CL	Centrolateral nucleus	4	4	26
PC	Paracentral nucleus	3	4	34
PaF	Parafascular nucleus	5	5	10
*Anterior group*				
AD	Anterodorsal nucleus	0	1	14
AV	Anteroventral nucleus	1	1	14
AM	Anteromedial nucleus	1	1	14
IAD	Interanterodorsal nucleus	3	2	2
LD	Laterodorsal nucleus	1	1	20
*Mediodorsal nucleus*				
MD	Mediodorsal nucleus	3	3	33
*Ventral group*				
VA	Ventral anterior nucleus	2	2	10
VL	Ventrolateral nucleus	2	2	18
VM	Ventromedial nucleus	2	2	28
VPM	Ventral posteromedial nucleus	2	2	32
VPL	Ventral posterolateral nucleus	1	2	36
VPPC	Ventral posterior parvicellular nucleus	3	3	12
Sub	Submedius nucleus	2	2	24
*Posterior group*				
LP	Lateral posterior nucleus	3	3	34
Po	Posterior nucleus	2	3	37
SG	Suprageniculate nucleus	3	4	6
*Geniculate nuclei*				
DLG	Dorsal lateral geniculate nucleus	2	1	23
MG	Medial geniculate nucleus	3	2	14
*Other*				
MH	Medial habenular nucleus	4	3	23
LH	Lateral habenular nucleus	1	2	19
PrG	Pregeniculate nucleus	1	1	24
Rt	Reticular nucleus	0	0	42
ZI	Zona incerta	0	0	40

**Table 3:** Summary of FoxP2 cell density and intensity levels and number of analyzed sections for each thalamic nucleus in macaques

	Thalamic nucleus	Density	Intensity	N
*Midline group*				
Pa	Paraventricular nucleus	5	4	20
Cs	Central superior nucleus	5	5	6
Cdc	Central nucleus densocellular part	4	3	10
Cim	Central nucleus intermediate part	4	3	9
Cif	Central nucleus inferior part	4	5	9
Clc	Central nucleus latocellular part	3	3	10
Ro	Rotundus nucleus	4	4	5
Re	Reuniens nucleus	3	3	16
Pt	Paratenial nucleus	4	4	5
*Intralaminar group*				
Csl	Central nucleus superior lateral part	4	3	9
Pcn	Paracentral nucleus	2	3	7
Cl	Central lateral nucleus	2	4	6
CnMd	Centromedian nucleus	3	5	11
Pf	Parafascicular nucleus	4	5	5
*Anterior group*				
AD	Anterodorsal nucleus	1	1	11
AV	Anteroventral nucleus	1	2	12
AM	Anteromedial nucleus	1	2	9
AI	Alaris nucleus	2	2	5
LD	Lateral dorsal nucleus	2	3	9
*Mediodorsal nucleus*				
MD	Mediodorsal nucleus	3	4	9
*Ventral group*				
VA	Ventral anterior nucleus	1	1	10
VL	Ventral lateral nucleus	2	2	13
VPL	Ventral posterior lateral nucleus	2	3	12
VPM	Ventral posterior medial nucleus	2	3	5
VPMpc	Ventral posterior medial nucleus, parvocellular part	3	3	6
VPI	Ventral posterior inferior nucleus	2	3	8
X	Area X	2	2	1
*Posterior group*				
SG	Suprageniculate nucleus	3	2	3
Li	Limitans nucleus	4	4	1
*Pulvinar group*				
Pul m	Medial pulvinar nucleus	3	3	3
Pul l	Lateral pulvinar nucleus	3	3	3
Pul i	Inferior pulvinar nucleus	4	3	4
Pul o	Oral pulvinar nucleus	4	2	3
LP	Lateral posterior nucleus	3	3	5
*Geniculate nuclei*				
GM	Medial geniculate nucleus	3	4	5
GLv	Ventral lateral geniculate nucleus	1	2	3
GLd	Dorsal lateral geniculate nucleus	5	1	11
*Other*				
Hm	Medial habenular nucleus	1	1	2
Hl	Lateral habenular nucleus	1	1	2
R	Reticular nucleus	0	0	25
ZI	Zona incerta	0	0	9

To enable cross-species comparisons, thalamic nuclei were grouped into the following groups: midline, intralaminar, anterior, ventral, and posterior groups, as well as the mediodorsal nucleus, the geniculate nuclei, and the pulvinar group (present only in macaques).

### Midline group

Across all species, Foxp2 expression was the highest in the midline group. In mice and rats, the paraventricular (PV), rhomboid (Rh) and xiphoid (Xi) nuclei showed the greatest cell density and labeling intensity. In macaques, the highest expression occurred in the paraventricular (Pa), central superior (Cs) and central inferior (Cif) nuclei. In mice, the lowest expression was observed in the interanteromedial (IAM) nucleus, with medium levels of cell density in the central medial (CM), reuniens (Re) and paraxiphoid (PaXi) nuclei and medium labeling intensity in the CM, Re and paratenial (PT) nuclei. In rats, Re and PaXi nuclei showed lower expression levels than in mice, although labeling intensity remained similar. In macaques, expression was generally high across all midline nuclei, with smaller variability between nuclei than in rodents. There was a slightly lower density in the Re and central latocellular (Clc) nuclei, and lower labeling intensity in the central densocellular (Cdc) and central intermedial (Cim) nuclei (Figs. 1 a1-c3, 2a1-c3).

### Intralaminar group

The intralaminar group showed the second highest Foxp2 expression across all species. Expression was strongest in the parafascicular PaF/Pf nucleus. In mice and rats, Foxp2 expression was higher in the centrolateral (CL) and PaF nuclei than in the paracentral (PC) and angular (Ang) nuclei, with labeling intensity generally higher in rats than in mice. In the macaque, expression density was high in the central superior lateral (Csl) and Pf nuclei, medium in the centromedian (CnMd) and lower in the paracentral (Pcn) and centrolateral (Cl) nuclei, while labeling intensity was the highest in the CnMd and Pf nuclei (Figs. 1 a1-c3, 2a1-c3).

### Anterior group

The anterior group showed the largest differences in Foxp2 expression across species. In mice, Foxp2 was detected only in the interanterodorsal (IAD) and laterodorsal (LD) nuclei, with relatively few labeled cells and low labeling intensity. A few cells were observed in the anteromedial (AM) nucleus, but their number did not reach the threshold to be considered very low expression. In rats, expression increased across anterior nuclei, with cells detected in the anteroventral (AV) and AM nuclei, and higher density and labeling intensity in the IAD nucleus. In macaques, FoxP2 was expressed throughout all anterior thalamic nuclei, with low to medium cell density and labeling intensity. Overall, Foxp2 expressions in the anterior nuclei increased progressively from mice to rats to macaques. The most notable difference was observed in the LD nucleus, which had low cell density and labeling intensity in mice and rats but medium–low density and medium intensity in macaques (Figs. 1a1-b3, 2 a1-b3).

### Mediodorsal nucleus

Foxp2 expression in the mediodorsal (MD) nucleus was largely conserved across all species studied. Cell density was similar in mice, rats, and macaques, at a medium level, while labeling intensity was slightly higher in macaques, reaching medium–high levels. Within the nucleus, Foxp2 labeling intensity appeared higher in the medial MD than in the lateral MD (Figs. 1b1-c3; 2b1-c3).

### Ventral group

The ventral group showed notable differences in Foxp2 expression across species, with both cell density and labeling intensity increasing from mice to rats and then to macaques. In mice, Foxp2 density was very low across all ventral nuclei, with virtually no expression in the ventral posteromedial (VPM). Labeling intensity was generally low but slightly higher in the ventral anterior (VA), ventrolateral (VL), and submedius (Sub) nuclei compared to other ventral nuclei. In rats, Foxp2 density and intensity were higher than in mice, with the ventral posterior parvicellular component (VPPC) nucleus showing the highest expression. In macaques, cell density was similar to that in rats, but labeling intensity was higher in most ventral nuclei, except for the VA nucleus, which exhibited low cell density and intensity (Figs. 1a1-d3; 2a1-d3).

### Posterior group

Foxp2 expression in the posterior group was generally similar between mice and rats, with slightly higher labeling intensity in the suprageniculate (SG) nucleus of rats. In macaques, SG cell density was conserved, but labeling intensity was lower. The limitans (Li) nucleus in macaques exhibited higher cell density and labeling intensity than the posterior nuclei of rodents. Foxp2 expression in the lateral posterior (LP) nucleus was consistent across all species, with similar cell density and labeling intensity (Figs. 1c1-2, d1-e2; 2c1-2, d1-e2).

### Pulvinar group

**Fig. 3:**
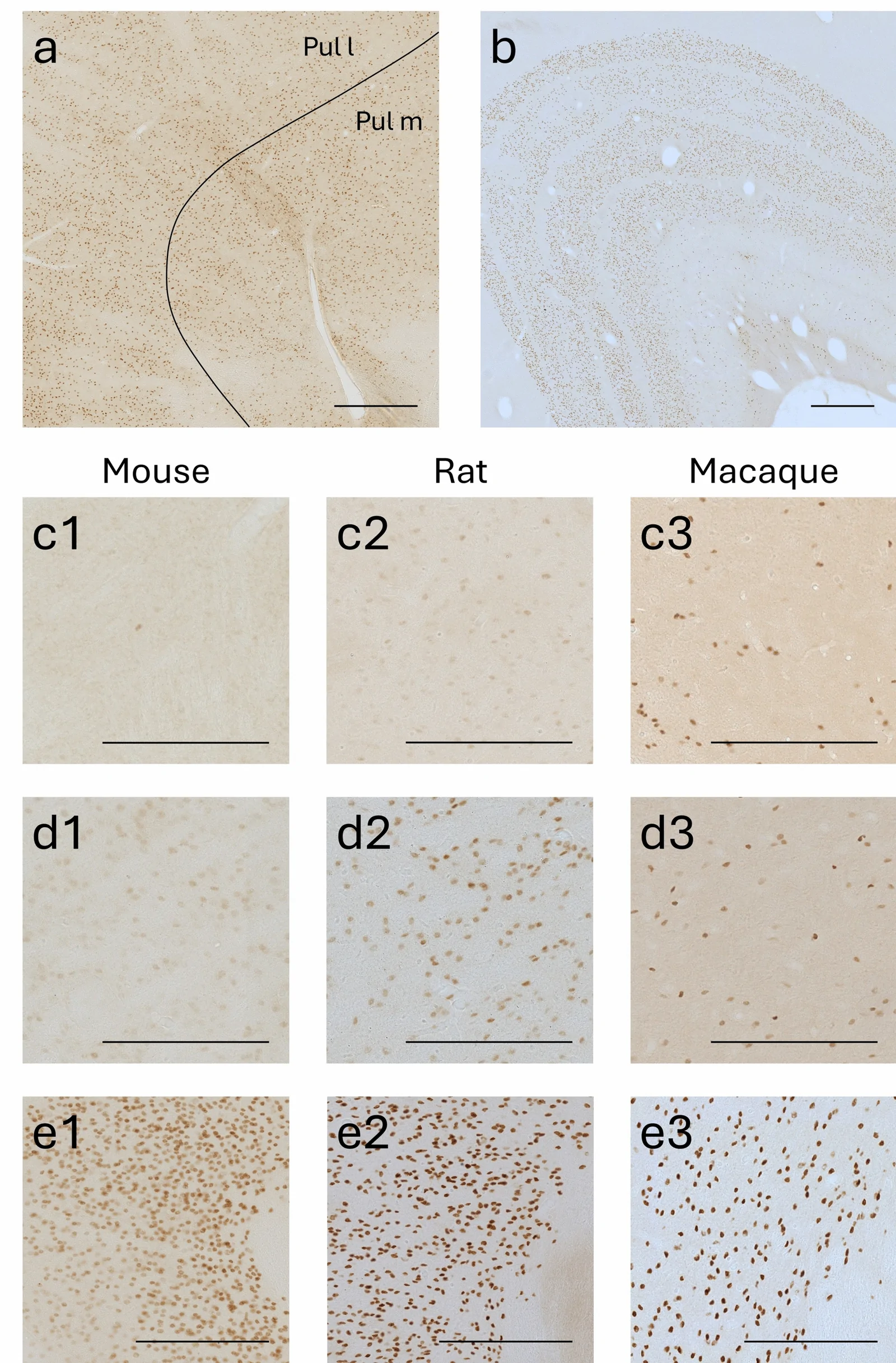
Representative images of Foxp2 labeling in the pulvinar (**a**) dorsal lateral geniculate (**b**) nuclei of macaque, and anteriomedial (**c**), ventral anterior (**d**) and parafascicular (**e**) nuclei of mouse (1), rat (2) or macaque (3). Scale bar: 500 μm (**a**, **b**), 250 μm (**c**, **e**)

Foxp2 is highly expressed in the pulvinar group in macaques, with the highest cell density observed in the pulvinar inferior (Pul i) and pulvinar oral (Pul o) nuclei, compared to lower density in the pulvinar medial (Pul m), pulvinar lateral (Pul l), and LP nuclei (Fig. 3a). Labeling intensity was relatively homogeneous across the pulvinar nuclei, with only a slightly lower intensity in Pul o (Figs. 1d3, e3; 2d3, e3).

### Geniculate nuclei

Foxp2 expression in the medial geniculate (MG) nucleus was largely conserved across species, with similar cell density, although labeling intensity was highest in macaques and lowest in rats. In contrast, expression in the dorsal lateral geniculate (DLG/GLd) nucleus showed pronounced species differences: mice and rats displayed relatively low Foxp2 expression, whereas macaques exhibited one of the highest levels. In macaques, FoxP2 expression was restricted to the parvocellular region, consistent with previous reports (Iwai et al. 2013) (Fig. 3b). Despite the high cell density in this region, likely influenced by cell size, labeling intensity remained relatively low. Expression in the macaque ventral lateral geniculate (GLv) nucleus was low with medium–low labeling intensity (Figs. 1c1-e2; 2c1-e2).

### Other

No Foxp2 expression was observed in the R/Rt nucleus or the ZI in any of the studied species, nor in the pregeniculate (PrG) nucleus of mice. Rats showed low expression in the PreG nucleus. In mice, Foxp2 was absent in the lateral habenula (LH) but showed medium–low expression in the medial habenula (MH). In rats, expression was low in the LH and medium–high in the MH. In both rodents, Foxp2-positive cells were concentrated in the lateral region of the MH. In macaques, both cell density and labeling intensity were low in the Hl and Hm (Figs. 1a1-e3; 2a1-e3).

In conclusion, Foxp2 is widely expressed in the thalamus of mice, rats and macaques with a wide range of Foxp2 + cell densities and intensities. From almost no Foxp2 expression in nuclei like AM (Fig. 3a1-a3), medium expression levels in VA (Fig. 3b1-b3) and high expression in PaF/Pf (Fig. 3c1-c3).

## Discussion

In this study, we provide a comparative analysis of Foxp2 expression across thalamic nuclei in mice, rats, and macaques. Expression was detected in most nuclei, with notable absence in the R/Rt nucleus and ZI, suggesting that Foxp2 is broadly involved in thalamic signaling but excluded from inhibitory regions. Foxp2 expression was particularly enriched in the midline and intralaminar nuclei, whereas the anterior thalamic group showed relatively weak labeling. Overall expression patterns were conserved between rodents and macaques, although several nuclei displayed species-specific differences. Macaques exhibited broader and, in some cases, more intense expression in the anterior, ventral, pulvinar, and lateral geniculate nuclei, regions associated with higher-order sensory and sensory-motor integration, except the lateral geniculate nucleus, which is a relay nucleus.

Foxp2 expression was the highest in the *midline group* across all studied species, particularly in the PV, Rh, and Xi nuclei of rodents and the PV, Cs and Cif nuclei of macaques. These nuclei are part of the limbic cortico-striato-thalamo-cortical loops (CSTCLs), projecting widely to the prefrontal and limbic cortex, hippocampus, and amygdala, and forming a central component of the limbic-thalamic network involved in memory and learning, attention, behavioral flexibility, and motivation (Joyce et al. 2022; Timbie et al. 2020; Vertes et al. 2015, 2022). Even though the midline nuclei have not directly been involved in language circuits, these processes are fundamental for supporting fluent language production and comprehension (Klostermann et al. 2013). The consistent high expression of Foxp2 in midline nuclei across the studied species suggests an association with thalamic circuits involved in these integrative functions.

The *intralaminar group* showed the second highest levels of Foxp2 expression, with the highest expression in the PaF/Pf and CnMd nuclei. The striatum, hippocampus, and cerebellum (limbic CSTCLs) are among the cortical and subcortical regions that these nuclei are closely linked to. Additionally, the PaF/Pf and CnMd project to visual, temporal, and frontal cortical regions (associative CSTCLs) (Eckert et al. 2011; Kumar et al. 2023). Thalamic aphasia has been linked to lesions in these nuclei, but there is evidence suggesting that these deficiencies may be caused by disturbances of pulvinar connection rather than damage to the thalamic nuclei themselves (Crosson 2013). In addition, these nuclei are involved in arousal, emotion, cognition, and motor processing, all of which may provide support for language functions (Kumar et al. 2023; Vertes et al. 2022). As in the midline nuclei, the elevated Foxp2 expression in the intralaminar nuclei is consistent with a potential involvement in the organization and maintenance of their connectivity. Additionally, the variations in Foxp2 levels among individual midline and intralaminar nuclei may reflect differences in their connectional and functional organization.

Foxp2 expression in the *mediodorsal* nucleus was moderate across species, with slightly higher levels in the macaques. This nucleus projects extensively to the prefrontal cortex helping support behavioral flexibility, adaptive decision-making, and memory (Mitchell 2015; Vertes et al. 2015) and participating in both limbic and associative CSTCLs. Lesions to the MD are not commonly linked to aphasia, although they affect verbal memory, causing anomia, perseveration, and executive deficits (Radanovic and Almeida 2021), indicating an indirect contribution to linguistic organization and control. Despite this, Crosson proposed that the MD nucleus's involvement in the cortico-striato-pallido-thalamo-frontal loops may contribute to linguistic procedural memory, which involves grammar (Crosson 2021b). All in all, Foxp2 expression in the MD-prefrontal circuit may be related to the modulation of synaptic efficiency and plasticity within networks supporting cognitive flexibility and adaptive behavior.

The *ventral group* showed notable differences across species, with Foxp2 levels increasing from rodents to macaques. The VA and VL have been found to be key structures in the sensorimotor cortico-striato-pallido-thalamo-frontal loops involved in motor speech production and fluency, as well as behavioral switching, timing, and sequential processing (Barbas et al. 2013). Lesions or stimulation of these nuclei cause reduced speech output and fluency, perseverations, and semantic paraphasias (Crosson 2021a; Johnson and Ojemann 2000). VA and VL have connections to the premotor and supplementary motor areas (Morel et al. 2005; Nakano et al. 1993). Additionally, VA is extensively connected to Broca's area (associative CSTCLs) (Bohsali et al. 2015). The increased expression of FoxP2 in macaques within these nuclei suggests differences in the organization of these thalamo-cortical circuits across species, particularly in regions associated with motor and associative processing.

The *pulvinar* nucleus displayed high FoxP2 expression in macaques. In humans, this nucleus, especially its medial region, is involved in lexical-semantic integration for correct word selection through its participation in associative cortico-thalamo-cortical circuits (Crosson 2021a, b). The Pul nucleus projects broadly to the cerebral cortex (Basile et al. 2024; Leh et al. 2008), including to Broca's area (Bohsali et al. 2015). Lesions in this region produce semantic paraphasias and fluent aphasia (Radanovic and Almeida 2021), while stimulation can transiently interrupt naming (Ojemann 1977). Thus, the pronounced FoxP2 expression in the macaque Pul is consistent with a potential role of Foxp2 in the modulation of cortico-thalamo-cortical interactions within associative networks.

Foxp2 expression was absent in the *reticular nucleus* and *zona incerta* across all species. The R/Rt nucleus regulates thalamic inhibition and attentional gating through GABAergic projections (Barbas et al. 2013; Crosson 2013). The lack of Foxp2 expression in this nucleus implies that its role is not in inhibitory modulation, but rather in the relay and associative nuclei that maintain excitatory thalamocortical projections.

The thalamic nuclei holding Foxp2 expression are part of cortico-striato-thalamo-cortical circuits involved in motor control, behavioral switching, timing, and sequential processing. The observed Foxp2 expression suggests a potential involvement in those functions. It is also relevant that Foxp2 is selectively highly expressed in the layer VI cortico-thalamic projection neurons (Hisaoka et al. 2010; Qi et al. 2024), which suggests that this transcription factor may be regulating both directions of the cortico-thalamo-cortical circuits, coordinating gene expression in neurons, and allowing the maintenance of the precision of the reciprocal communication. Different studies support this hypothesis, as disruptions of Foxp2 expression in the cortex, striatum, cerebellum, and thalamus affect the connectivity and plasticity of cortico-striatal, cerebellar, and thalamo-striatal circuits (Chen et al. 2016; French et al. 2019; Rodríguez-Urgellés et al. 2023). Through its role as a transcription factor regulating neurogenesis, synaptic plasticity, and axonal guidance (den Hoed et al. 2021), Foxp2 may be contributing to the fine-tuning of these circuits, ensuring the necessary temporal precision and cross-modal integration of information across cortical, subcortical, and cerebellar components of speech-related networks.

FoxP2 expression studies performed in other mammals, such as echolocating bats (Rodenas-Cuadrado et al. 2018) confirm a great conservation of Foxp2 across the mammalian thalamic nuclei. In addition, studies in vocal-learning birds show that FoxP2 expression is, at least, partially conserved between birds and mammals. FoxP2 is highly expressed in the *nucleus dorsolateralis anterior thalami, pars medialis*, which is homologous to parts of the midline, intralaminar, and mediodorsal thalamic nuclei. It is also highly expressed in the ventrointermediate area, homologous to the ventral thalamic group. Furthermore, FoxP2 is expressed in the nucleus ovoidalis, which is involved in auditory input, as well as in the nucleus rotundus and *nucleus superficialis parvocellularis*, which are involved in visual processing (Haesler et al. 2004; Teramitsu et al. 2004). In this context, the conserved expression of FoxP2 in homologous thalamic nuclei across mammals and vocal-learning birds supports the idea that Foxp2 might be playing a role in the evolutionarily conserved cortico-striato-thalamo-cortical circuits underlying sensorimotor integration and learned vocal behavior.

No studies have been performed in the human adult thalamus; although, in the human fetal thalamus, FOXP2 has a similar expression pattern as in the macaque, with moderate-high FOXP2 expression in the pulvinar nucleus, high expression in the intralaminar, midline, and ventral nuclei, medium expression in the MD and low to no expression in the anterior thalamic nuclei (Alhesain et al. 2025; Teramitsu et al. 2004). The most relevant finding of these studies is a high expression in the VA nucleus (Alhesain et al. 2025), which has a relatively low Foxp2 expression in the species studied in the present study. This reinforces the idea that during human evolution, FOXP2 expression has become selectively enhanced in thalamic territories associated with speech motor control and language processing. Given the role of the VA nucleus in the cortico-striato-pallido-thalamo-frontal loops (Barbas et al. 2013) and its dense connectivity with Broca's and premotor areas (Bohsali et al. 2015), this enhancement may reflect a molecular adaptation supporting the evolution of complex vocal behaviors and the modulation of motor-cognitive integration required for language.

One of the main differences between the primate (including human) thalamus and that of rodents is the selective expansion of associative nuclei, such as the pulvinar and mediodorsal nuclei, as well as the increased complexity and refinement of thalamo-cortical projections (Ruiz-Cabrera et al. 2023). These structural enlargements parallel the evolutionary expansion of association cortices and the emergence of higher-order cognitive and communicative abilities in primates. Foxp2 might have contributed to these evolutionary changes, as it is highly expressed in associative nuclei. Experimental studies in mice have shown that Foxp2 expression manipulation affects thalamic patterning. Foxp2 knock-out or R552H knock-in mutants exhibit a reduction of posterior thalamic regions and an expansion of intermediate nuclei (Ebisu et al. 2017), and similar reductions in thalamic volume have been observed in patients with *FOXP2* mutations (Belton et al. 2003; Liégeois et al. 2016). Moreover, studies introducing humanized FOXP2 have found increased dendritic length of specifically thalamic, striatal, and layer VI pyramidal neurons (Enard et al. 2009; Reimers-Kipping et al. 2011), highlighting its role in the evolutionary reorganization of cortico-basal ganglia and cortico-thalamic circuits.

Beyond evolution, these same molecular pathways may also confer vulnerability to neurodevelopmental and psychiatric disorders. Thalamocortical dysconnectivity is a common hallmark for of these conditions, including schizophrenia, bipolar disorder, major depressive disorders (MDD), autism spectrum disorder (ASD), and attention deficit and hyperactivity disorder (ADHD) (Giraldo-Chica & Woodward 2017; Nair et al. 2015; Sheffield et al. 2020; Shi et al. 2025; Tomasi & Volkow 2019; Tu et al. 2019). In these patients there is usually a reduction in thalamo-prefrontal connectivity and an increased thalamo-somatosensory connectivity. FOXP2 expression has been found to be reduced in schizophrenia and ASD patients, correlating with grey matter loss and cognitive and executive function deficits, respectively (Haghighatfard et al. 2022; Sanjuán et al. 2021). Moreover, the polygenic risk score of *FOXP2*-related genes has been strongly associated with functional dysconnectivity identified in schizophrenia patients with shorter illness duration, which in turn correlates with symptom score (Du et al. 2021). Our results in mice, rats, and macaques show that Foxp2 expression is particularly enriched in associative nuclei with strong thalamocortical connectivity to prefrontal and association cortices, the same circuits that, in humans, are disrupted in these disorders. A reduction or dysregulation of FOXP2 expression in humans could alter the connectivity of these networks, leading to the language, executive, and attentional impairments seen in these conditions. Thus, understanding Foxp2 expression in the thalamus not only informs us how language and cognitive abilities evolved but also how their dysfunctions can lead to disorders.

While these findings shed light on the potential role of Foxp2 in shaping thalamic circuits relevant to language and cognition, several limitations of this study should be considered. First, due to technical constraints we were unable to examine Foxp2 expression in adult human thalamic tissue. Current information is restricted to early human development (Alhesain et al. 2025; Teramitsu et al. 2004), although evidence from other species suggests that thalamic Foxp2 expression patterns established early in life are largely preserved into adulthood (Ferland et al. 2003; Takahashi et al. 2008). Second, the relationship between Foxp2 expression levels and the establishment and function of thalamo-cortical circuits remains correlational. Further research combining manipulations of Foxp2 expression in vivo and axon-tracing would be necessary to determine how it translates into connectivity and circuit function. Additionally, the limited sample size and the composition of the primate cohort, which included two macaque species and only one female animal, prevent the assessment of species- or sex-related differences in FoxP2 expression. Although previous studies have reported limited sexual dimorphism in FoxP2 expression across several vertebrate species (Campbell et al. 2009; Teramitsu et al. 2004), future studies including larger and sex-balanced cohorts will be necessary to address this question directly. Finally, as language is a human-specific capacity, the specific contribution of individual thalamic nuclei to language circuits remains poorly defined, limiting our ability to infer the precise role of Foxp2 in these pathways.

In summary, Foxp2 is broadly expressed in the thalamus of rodents and primates, with conserved high expression in the midline and intralaminar nuclei and enhanced expression in associative thalamic nuclei in primates. This distribution mirrors the organization of thalamocortical circuits involved in complex cognition, suggesting that Foxp2 may contribute to the molecular tuning of the circuits supporting those funcions, including language and speech.

## Supplementary Information

Below is the link to the electronic supplementary material.

Supplementary file1 (XLSX 22 KB)

Supplementary file2 (IJM 2 KB)

## Data Availability

No datasets were generated or analysed during the current study.
